# S Protein-Reactive IgG and Memory B Cell Production after Human SARS-CoV-2 Infection Includes Broad Reactivity to the S2 Subunit

**DOI:** 10.1128/mBio.01991-20

**Published:** 2020-09-25

**Authors:** Phuong Nguyen-Contant, A. Karim Embong, Preshetha Kanagaiah, Francisco A. Chaves, Hongmei Yang, Angela R. Branche, David J. Topham, Mark Y. Sangster

**Affiliations:** a David H. Smith Center for Vaccine Biology and Immunology, Department of Microbiology and Immunology, University of Rochester Medical Center, Rochester, New York, USA; b Department of Biostatistics and Computational Biology, University of Rochester Medical Center, Rochester, New York, USA; c Department of Medicine, University of Rochester Medical Center, Rochester, New York, USA; Washington University School of Medicine; St. Jude Children's Research Hospital

**Keywords:** COVID-19, IgG antibodies, memory B cells, SARS-CoV-2, spike protein

## Abstract

The high susceptibility of humans to severe acute respiratory syndrome coronavirus 2 (SARS-CoV-2) infection, the cause of coronavirus disease 2019 (COVID-19), reflects the novelty of the virus and limited preexisting B cell immunity. IgG against the SARS-CoV-2 spike (S) protein, which carries the novel receptor binding domain (RBD), is absent or at low levels in unexposed individuals. To better understand the B cell response to SARS-CoV-2 infection, we asked whether virus-reactive memory B cells (MBCs) were present in unexposed subjects and whether MBC generation accompanied virus-specific IgG production in infected subjects. We analyzed sera and peripheral blood mononuclear cells (PBMCs) from non-SARS-CoV-2-exposed healthy donors and COVID-19 convalescent subjects. Serum IgG levels specific for SARS-CoV-2 proteins (S, including the RBD and S2 subunit, and nucleocapsid [N]) and non-SARS-CoV-2 proteins were related to measurements of circulating IgG MBC levels. Anti-RBD IgG was absent in unexposed subjects. Most unexposed subjects had anti-S2 IgG, and a minority had anti-N IgG, but IgG MBCs with these specificities were not detected, perhaps reflecting low frequencies. Convalescent subjects had high levels of IgG against the RBD, S2, and N, together with large populations of RBD- and S2-reactive IgG MBCs. Notably, IgG titers against the S protein of the human coronavirus OC43 were higher in convalescent subjects than in unexposed subjects and correlated strongly with anti-S2 titers. Our findings indicate cross-reactive B cell responses against the S2 subunit that might enhance broad coronavirus protection. Importantly, our demonstration of MBC induction by SARS-CoV-2 infection suggests that a durable form of B cell immunity is maintained even if circulating antibody levels wane.

## Abstract (precis)

The recent rapid worldwide spread of SARS-CoV-2 has established a pandemic of potentially serious disease in the highly susceptible human population. Key issues are whether humans have preexisting immune memory that provides some protection against SARS-CoV-2 and whether SARS-CoV-2 infection generates lasting immune protection against reinfection. Our analysis focused on pre- and postinfection IgG and IgG memory B cells (MBCs) reactive to SARS-CoV-2 proteins. Most importantly, we demonstrate that infection generates both IgG and IgG MBCs against the novel receptor binding domain and the conserved S2 subunit of the SARS-CoV-2 spike protein. Thus, even if antibody levels wane, long-lived MBCs remain to mediate rapid antibody production. Our study results also suggest that SARS-CoV-2 infection strengthens preexisting broad coronavirus protection through S2-reactive antibody and MBC formation.

## INTRODUCTION

The betacoronavirus severe acute respiratory syndrome coronavirus 2 (SARS-CoV-2), the causative agent of a respiratory disease termed coronavirus disease 2019 (COVID-19), emerged in China in late 2019 and rapidly spread worldwide (1). A pandemic was declared in March 2020, and global deaths from COVID-19 now exceed 900,000. The rapid increase in cases in many countries has challenged health care systems, and shutdowns and quarantine measures introduced to slow virus spread have caused major disruptions to society and economies (2). SARS-CoV-2 infection produces a wide spectrum of outcomes. A proportion of infections, likely more than 20%, remain asymptomatic. Most clinical cases develop mild to moderate respiratory symptoms, but up to 20% progress to a more severe disease with extensive pneumonia (3, 4).

When SARS-CoV-2 emerged and began to spread, the severity of the threat was primarily attributed to the novelty of the virus to the human immune system and, consequently, a lack of preexisting immune memory to quickly clear virus and limit disease progression. Four types of common cold coronavirus are endemic in humans, including the alphacoronaviruses 229E and NL63 and the betacoronaviruses OC43 and HKU1. However, limited relatedness between key structural proteins of these human coronaviruses (HCoVs) and those of SARS-CoV-2 suggested that significant cross-reactive immunity was unlikely (5, 6). Initial studies of non-SARS-CoV-2-exposed individuals found negligible levels of IgG against the SARS-CoV-2 spike (S) protein, the viral attachment protein that binds receptor angiotensin converting enzyme 2 (ACE2) on host cells to initiate infection (7). More recently, however, studies have provided evidence of SARS-CoV-2-reactive B and T cell memory in unexposed subjects that could confer some protection against SARS-CoV-2 or modulate disease pathogenesis (8–10).

Sera from non-SARS-CoV-2-exposed individuals have been screened for IgG binding to the S1 and S2 subunits of the SARS-CoV-2 S protein. The membrane-distal S1 subunit contains the receptor binding domain (RBD) for receptor recognition, and the membrane-proximal S2 subunit, which has higher homology among coronaviruses than does S1 (6, 8), mediates membrane fusion to release viral RNA into the host cell. In two large cohorts of unexposed subjects, approximately 10% had IgG that bound S2 but not S1 or the RBD. Approximately 4% of the subjects had IgG against the SARS-CoV-2 nucleocapsid (N) protein, which is highly conserved among coronaviruses (10, 11). Although N is an internal viral protein and not a target of neutralizing antibodies (Abs), coronavirus infections typically elicit strong anti-N Ab production (12). The idea that circulating HCoVs elicit IgG that cross-reacts with SARS-CoV-2 is supported by the finding that SARS-CoV-2 infection increases IgG titers against the S proteins of multiple HCoVs (13). In T cell studies, CD4^+^ T cells in up to 50% of non-SARS-CoV-2-exposed donors responded to epitopes in S and non-S proteins of SARS-CoV-2 (8, 9). Notably, S-reactive CD4^+^ T cells in unexposed subjects were mostly reactive to the conserved S2 subunit, consistent with cross-reactivity to circulating HCoVs (8). SARS-CoV-2-reactive CD8^+^ T cells were also detected in unexposed donors, but the response was less marked than for CD4^+^ T cells (9).

SARS-CoV-2-reactive memory B cells (MBCs) generated in B cell responses to HCoVs are also likely to be present in non-SARS-CoV-2-exposed individuals. Indeed, MBCs might be more important than preexisting cross-reactive Abs as a source of protection against SARS-CoV-2. IgG MBCs are more broadly reactive than bulk serum Abs generated against the same antigen, they persist after circulating Ab levels wane, and they are readily activated to generate strong Ab responses or seed germinal centers for additional rounds of affinity maturation (14). Concurrent early production of virus-specific IgM and IgG in the response to SARS-CoV-2 infection suggests a response mediated by IgG MBCs as well as by naive B cells (10, 15–17). This picture is supported by identification of B cell subsets with high and low immunoglobulin V gene mutation frequencies during the response to SARS-CoV-2 infection (18). However, little direct analysis of SARS-CoV-2-reactive MBCs in unexposed subjects has been performed.

Characterization of populations of MBCs generated and/or expanded by SARS-CoV-2 infection can also provide insights into cross-reactivity between coronaviruses and participation of preexisting MBCs in the response. Wec et al. (19) used cells from a survivor of the 2003 SARS-CoV outbreak as a source of MBCs that bound the S protein of SARS-CoV-2; a comprehensive panel of Abs expressed by the MBCs were cloned and characterized. Notably, most of the highly mutated MAbs bound the S2 subunit of multiple HCoV S proteins, often with higher affinity than to the S2 of SARS-CoV-2. A screening of healthy donors identified low frequencies of MBCs reactive to the S proteins of the 2003 SARS-CoV and SARS-CoV-2 (19). Findings suggest that S2-reactive MBCs generated by HCoVs were activated and expanded by the 2003 SARS-CoV. RBD-binding MBCs sampled in the convalescent phase of SARS-CoV-2 infection expressed Abs with relatively low numbers of V gene mutations, suggesting that this component of the response largely reflected naive B cell activation by novel epitopes (20).

To extend our understanding of the B cell response to SARS-CoV-2 infection, the current study compared Ab and MBC immunities to SARS-CoV-2 in unexposed individuals and individuals in the convalescent phase of infection. In particular, we were interested in the presence of SARS-CoV-2-reactive MBCs in unexposed subjects that could confer some protection against SARS-CoV-2 and in formation of MBCs by SARS-CoV-2 infection to provide durable protection against reinfection. Most importantly, we demonstrate that SARS-CoV-2 infection generates both IgG and IgG MBCs reactive to the novel RBD and the conserved S2 subunit of the S protein. Long-lived MBCs are thus likely to be available to mediate rapid protective Ab responses if circulating Ab levels wane and reinfection occurs. Our results also draw attention to preexisting SARS-CoV-2-cross-reactive B cell memory corresponding to the S2 subunit in SARS-CoV-2-naive subjects. We speculate that the strong response to S2 after SARS-CoV-2 infection reflects preexisting S2-reactive MBC activation and strengthens broad coronavirus protection.

## RESULTS

### IgG against SARS-CoV-2 proteins in unexposed subjects primarily targets the S2 subunit of the S protein.

To investigate preexisting B cell immunity to SARS-CoV-2 in unexposed individuals and SARS-CoV-2-reactive B cell immunity generated by infection, we analyzed sera and peripheral blood mononuclear cells (PBMCs) from (i) 21 healthy donors sampled prior to the emergence of SARS-CoV-2 and (ii) 26 nonhospitalized COVID-19 convalescent subjects sampled 4 to 9 weeks after symptom onset. Reactivity was measured against the S protein (including the RBD and S2 subunit) and N protein of SARS-CoV-2 and the S proteins of the human alphacoronavirus 229E and betacoronavirus OC43. H1 influenza virus hemagglutinin and tetanus toxoid (TTd) were included as control antigens that humans are commonly exposed to through infection and vaccination.

**FIG 1:**
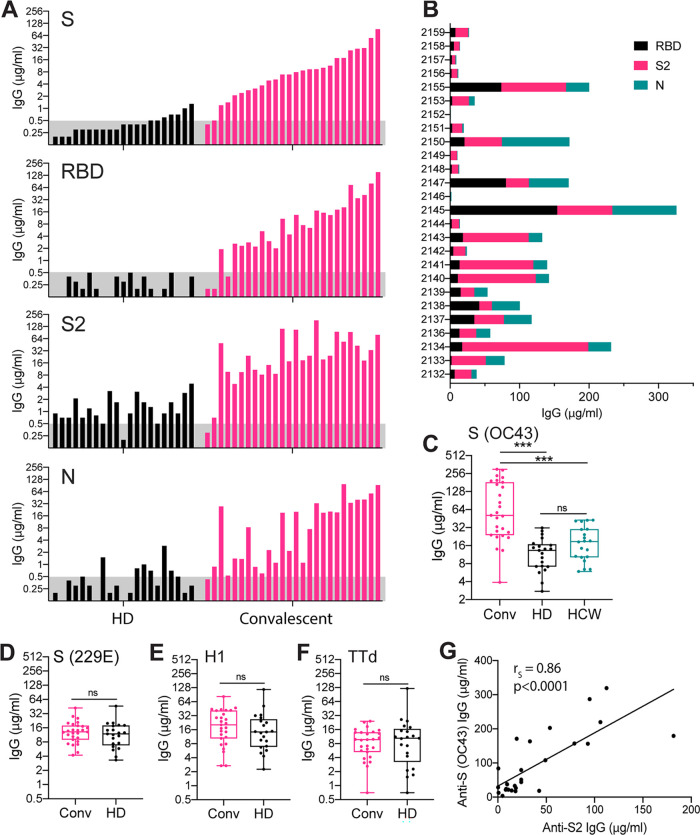
Serum IgG levels against SARS-CoV-2 and non-SARS-CoV-2 proteins in non-SARS-CoV-2-exposed and COVID-19 convalescent subjects. Sera were collected from (i) 21 healthy donors sampled from 2011 to 2014 (HD), (ii) 20 SARS-CoV-2-negative health care workers sampled in 2020 (HCW), and (iii) 26 COVID-19 convalescent subjects sampled 4 to 9 weeks after symptom onset (Conv). (A) Serum IgG concentrations measured by ELISA against the SARS-CoV-2 spike (S), receptor binding domain (RBD), S2 subunit, and nucleocapsid (N). Columns represent individual HD and convalescent subjects in order of ascending titers against S. The assigned cutoff for positivity is shown by the horizontal gray bar. (B) Proportions of serum IgG against SARS-CoV-2 RBD, S2, and N for individual convalescent subjects. (C) Serum IgG concentrations against the S protein of the HCoV OC43 in Conv, HD, and HCW subjects. (D to F) Serum IgG concentrations against the S protein of HCoV 229E (D), influenza virus H1 hemagglutinin (E), and TTd (F) in Conv and HD subjects. (G) Correlation between serum IgG concentrations against the S2 subunit of SARS-CoV-2 and the S protein of the HCoV OC43. Significance (*, *P* < 0.05; **, *P* < 0.01; ***, *P* < 0.001; ns, not significant) for comparisons of serum IgG concentrations between subject groups was determined by the Wilcoxon rank sum test. Correlations were tested by Spearman correlation analysis with corresponding robust regression models.

Serum IgG levels were measured by enzyme-linked immunosorbent assay (ELISA). Approximately one-third of non-SARS-CoV-2-exposed subjects in the healthy donor cohort had low levels of serum IgG against the S and N proteins of SARS-CoV-2, likely reflecting cross-reactivity with seasonal HCoVs (Fig. 1A). Notably, 86% of unexposed subjects had IgG against the highly conserved S2 subunit of the S protein. It is possible that inherent features of the bulky S reagent used in our analysis reduced binding by anti-S2 Abs. IgG that bound the highly novel RBD was not detected in unexposed subjects. All non-SARS-CoV-2-exposed subjects had IgG against S proteins of HCoVs 229E and OC43, indicating previous infection, and against control proteins H1 and TTd (Fig. 1C to F).

### S- and N-specific IgG production following SARS-CoV-2 infection includes a strong response to the S2 subunit.

Levels of IgG against S, RBD, S2, and N were markedly higher in convalescent subjects than in unexposed subjects, indicating strong induction of these Abs by SARS-CoV-2 infection (Fig. 1A). In a lower number of convalescent subjects, high anti-S IgG titers were associated with low levels of anti-N IgG. Indeed, more than 40% of convalescent subjects had anti-N IgG levels within the range seen in unexposed subjects, questioning the reliability of using anti-N IgG measurement to identify previous SARS-CoV-2 infection in recovered patients (21).

Notably, serum IgG titers against S2 were consistently higher than against the RBD in convalescent subjects, perhaps reflecting the novelty of the RBD and a response dependent on naive B cell activation (Fig. 1B). Interestingly, titers of IgG were higher against the S protein of the HCoV OC43 in convalescent subjects than in unexposed subjects, but this was not the case for the S protein of HCoV 229E (or for the control proteins H1 and TTd) (Fig. 1C to F). The anti-OC43 S IgG titers correlated with those against the SARS-CoV-2 S (*r_S_* = 0.49, *P = *0.0109), RBD (*r_S_* = 0.57, *P* = 0.0025), and S2 (*r_S_* = 0.86, *P < *0.0001), indicating a relationship with SARS-CoV-2 infection (Fig. 1G). The particularly strong correlation between IgG titers against OC43 S and the SARS-CoV-2 S2 suggests a cross-reactive response to the S2 subunit.

Since the healthy donor samples in our analysis were collected 6 to 10 years before the emergence of SARS-CoV-2, we considered the possibility that a recently circulating HCoV was responsible for the higher anti-OC43 S IgG titers in the convalescent subjects. To exclude this possibility, we measured anti-OC43 S IgG titers in sera collected from 20 health care workers in 2020. The health care workers had cared for hospitalized SARS-CoV-2 patients, but all were negative for IgG against SARS-CoV-2 S and RBD, consistent with the effectiveness of personal protective equipment and appropriate work practices. OC43 S-reactive IgG levels in health care worker sera were similar to those in non-SARS-CoV-2-exposed healthy donor sera and significantly lower than those in sera from convalescent subjects (Fig. 1C). Taken together, our results indicate that SARS-CoV-2 infection generates a strong IgG response that cross-reacts with the S2 of human betacoronaviruses.

### Strong S-reactive MBC formation following SARS-CoV-2 infection includes reactivity to the RBD and S2 subunit.

PBMCs from non-SARS-CoV-2-exposed subjects and convalescent subjects were analyzed for the presence of MBCs reactive to SARS-CoV-2 proteins. Circulating antigen-specific IgG MBC populations were measured by *in vitro* stimulation of MBCs to induce differentiation into Ab-secreting cells (ASCs). Poststimulation antigen-specific measurement of levels of MBC-derived ASCs (MASCs) by enzyme-linked immunosorbent spot (ELISpot) assay or of MBC-derived polyclonal Abs (MPAbs) by ELISA provided a measure of the levels of precursor MBCs (22). Analysis of MASCs by ELISpot assay was performed against the SARS-CoV-2 S, RBD, and N proteins and against influenza virus H1 and TTd. MPAb levels were measured against those of antigens used in the ELISpot assay, as well as SARS-CoV-2 S2 and the S proteins of HCoVs OC43 and 229E. Antigen-specific IgG MPAb concentrations correlated strongly with the frequency of IgG MASCs derived from stimulated MBCs (determined for SARS-CoV-2 S, SARS-CoV-2 RBD, influenza virus H1, and TTd, *r_S_* = 0.89, 0.67, 0.83, and 0.95, respectively, *P ≤ *0.0002), validating the use of the MPAb concentration as a measure of the size of specific MBC populations.

**FIG 2:**
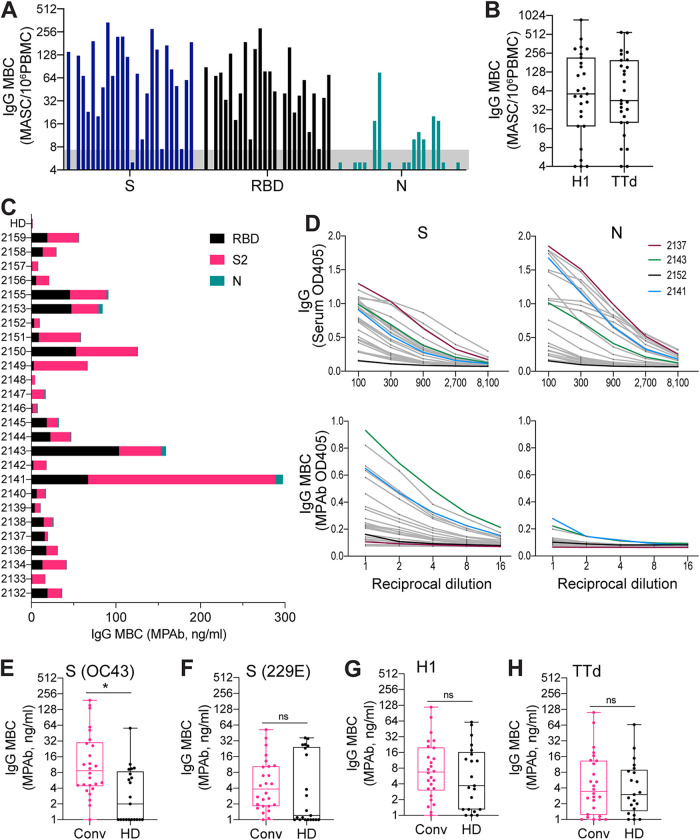
Analysis of IgG memory B cells (MBCs) reactive to SARS-CoV-2 and non-SARS-CoV-2 proteins in non-SARS-CoV-2-exposed and COVID-19 convalescent subjects. PBMCs for MBC analysis were collected from (i) 21 healthy donors sampled from 2011 to 2014 (HD) and (ii) 26 COVID-19 convalescent subjects sampled 4 to 9 weeks after symptom onset (Conv). PBMCs were stimulated *in vitro* to induce MBC differentiation into Ab-secreting cells. Antigen-specific quantitation of MBC-derived Ab (IgG)-secreting cells (MASCs) by ELISpot assay or of MBC-derived polyclonal (IgG) Abs (MPAbs) by ELISA provided a measure of the abundance of specific IgG MBCs. (A) IgG MBCs reactive to the SARS-CoV-2 spike (S), receptor binding domain (RBD), and nucleocapsid (N) in convalescent subjects. The assigned cutoff for positivity is shown by the horizontal gray bar. (B) IgG MBCs reactive to the influenza virus H1 hemagglutinin and TTd in convalescent subjects. (C) Proportions of IgG MBCs reactive to the SARS-CoV-2 RBD, S2, and N for individual convalescent subjects. A bar representing the mean value for the HD cohort is included for comparison. In all HD samples, MPAb IgG levels against RBD, S2, and N were below the cutoff for assay positivity. (D) Comparison of serum IgG concentrations (upper panels) and numbers of IgG MBCs (lower panels) reactive to the SARS-CoV-2 S (left-hand side) and N (right-hand side) proteins. Serum IgG was measured by ELISA; IgG MBC numbers were based on ELISA of MPAbs. Dilution curves are shown for individual convalescent subjects; curves for 4 subjects are shown in different colors to identify particular response patterns. (E to H) IgG MBCs reactive to the S proteins of HCoVs OC43 (E) and 229E (F), the H1 hemagglutinin (G), and TTd (H) in convalescent and HD subjects. Significance (*, *P < *0.05; ns, not significant) for comparisons of IgG MBC numbers between subject groups was determined by the Wilcoxon rank sum test.

The presence of a low level of IgG against the SARS-CoV-2 S, RBD, and N proteins in a proportion of unexposed subjects suggested that IgG MBCs with the same specificity had also been formed. However, these MBCs were not detected (Fig. 2C), possibly because of very low frequencies in the circulation. In contrast, IgG MBCs reactive to the S proteins of the HCoVs OC43 and 229E and the control proteins H1 and TTd were detected in nearly 50% or more of non-SARS-CoV-2-exposed subjects, consistent with the higher levels of serum IgG against these antigens (Fig. 2E to H). As expected, SARS-CoV-2 RBD-reactive MBCs were not detected in unexposed subjects.

In marked contrast to non-SARS-CoV-2-exposed subjects, the vast majority of convalescent subjects had circulating IgG MBCs reactive to SARS-CoV-2 S, RBD, and S2, indicating strong induction by SARS-CoV-2 infection of MBCs reactive to novel and conserved regions of the S protein (Fig. 2A and C). Notably, numbers of IgG MBCs reactive to the S protein of the HCoV OC43 were higher in convalescent subjects than in unexposed subjects (Fig. 2E), but there was no difference between the two subject groups in the levels of IgG MBCs reactive to the HCoV 229E S protein or influenza virus H1 or TTd (Fig. 2B and F to H). S2-reactive IgG MBC numbers correlated well with levels of IgG MBCs reactive to SARS-CoV-2 S (*r_S_* = 0.77, *P < *0.0001) and RBD (*r_S_* = 0.60, *P = *0.0012) and to S of HCoV OC43 (*r_S_* = 0.52, *P = *0.0059) but not with those reactive to S of HCoV 229E (*r_S_* = −0.13, *P = *0.53), influenza virus H1 (*r_S_* = 0.13, *P = *0.54), or TTd (*r_S_* = 0.29, *P = *0.15). The findings of our MBC analysis are consistent with serum IgG measurement and indicate that SARS-CoV-2 infection generates IgG MBCs reactive to the SARS-CoV-2 S2 that cross-react with the S2 of human betacoronaviruses. Interestingly, only a small proportion of the convalescent subjects generated detectable N-reactive IgG MBCs, even though most subjects produced high levels of anti-N IgG in serum (Fig. 2C and D). It is unclear whether this reflects a real difference between S-reactive MBC formation and N-reactive MBC formation or an effect of the sampling time. Overall, we demonstrate that SARS-CoV-2 infection induces strong S-reactive MBC formation that would be expected to provide lasting protection against reinfection and, potentially, broad protection against betacoronaviruses.

## DISCUSSION

Our goals in this study were to investigate SARS-CoV-2-reactive B cell memory in unexposed subjects that could provide some protection against SARS-CoV-2 infection and the generation of B cell memory by SARS-CoV-2 infection that could provide lasting protection against reinfection. In particular, we were interested in IgG MBCs, which respond to cognate antigens with rapid, vigorous, and high-affinity Ab production. Importantly, MBCs are long-lived cells that continue to provide strong protection when circulating Ab levels wane. Our approach was to analyze circulating IgG as well as IgG MBCs from the SARS-CoV-2-naive and SARS-CoV-2-convalescent subject groups. Our key findings are as follows: (i) the presence of IgG reactive to the S2 subunit of SARS-CoV-2 in most unexposed subjects, likely reflecting cross-reactivity to HCoVs; (ii) markedly increased levels of IgG against the SARS-CoV-2 S and N proteins, including reactivity to the RBD and S2 subunit of S, in convalescent subjects; (iii) increased IgG binding to the S protein of the OC43 HCoV, but not the 229E HCoV, in convalescent subjects, reflecting greater cross-reactivity between S2 subunits of betacoronaviruses; (iv) strong formation of IgG MBCs reactive with the RBD and S2 subunit of the SARS-CoV-2 S protein in convalescent subjects; and (v) formation of IgG MBCs reactive with the S protein of OC43, but not with that of 229E, in convalescent subjects, consistent with S2 subunit cross-reactivity between betacoronaviruses.

Approximately one-third of our cohort of non-SARS-CoV-2-exposed subjects had low levels of IgG against the SARS-CoV-2 S and N proteins. The low anti-N IgG level likely reflects infection with HCoVs, which have low-level (20% to 30%) homology with the SARS-CoV-2 N protein (11). However, a protective function for anti-N Abs has not been established (23). Notably, 86% of unexposed subjects had IgG against the S2 subunit, reflecting homology with HCoVs, but none had IgG against the highly novel SARS-CoV-2 RBD (6, 8, 24). Abs that target the S2 subunit have been shown to have virus-neutralizing activity, raising the possibility that the presence of preexisting anti-S2 IgG confers some protection against SARS-CoV-2 (25). The processes that generate anti-S2 IgG are also likely to generate S2-reactive IgG MBCs, and these might provide more significant protection than low levels of anti-S2 Abs. However, S2-reactive MBCs (or S-reactive and N-reactive MBCs) were not detected in non-SARS-CoV-2-exposed subjects. Taking those findings together with the identification of S-reactive MBCs in unexposed healthy donors (19), it is likely that the levels of S2-reactive MBCs were below the limit of detection in our assays. On the basis of an estimate of 10 to 20 IgG MASCs generated per IgG MBC after *in vitro* stimulation (26), our analysis suggests that the frequency of S2-reactive MBCs, if present in unexposed healthy donors, would be <1/10^6^ PBMCs. Most MBCs are resident in lymphoid tissues and, except for MBCs against frequently seen immunogenic antigens (for example, the influenza virus H1 or TTd in this study), are at very low frequencies in the circulation in the steady state (27, 28).

Anti-RBD, anti-S, and anti-N IgG levels were markedly higher in the convalescent subjects than in non-SARS-CoV-2-exposed subjects, indicating strong induction by SARS-CoV-2 infection. Perhaps notably, the majority of convalescent subjects had higher IgG titers against the S2 than against the RBD. This is particularly surprising because of the accessibility of the RBD to B cells and the expected immunodominance over the S2 subunit (29, 30). Our demonstration of strong anti-S2 IgG production is consistent with the activation of a preexisting population of IgG MBCs against the conserved S2 subunit in the absence of MBCs reactive to the novel RBD. However, we cannot exclude the possibility of inherent differences in the stability or antigenicity of RBD and S2 reagents as an explanation. IgG levels against the S protein of HCoV OC43 (but not 229E) were significantly higher in convalescent subjects than in non-SARS-CoV-2-exposed subjects and correlated strongly with anti-S2 IgG levels. These findings support the idea of stronger B cell cross-reactivity between the S2 subunits of SARS-Cov-2 and human betacoronaviruses than alphacoronaviruses (8).

Importantly, we demonstrated that SARS-CoV-2 infection generates RBD-reactive and S2-reactive IgG MBCs. Recently, Long et al. (4) found that levels of SARS-CoV-2-reactive Abs, including neutralizing Abs, start to decrease within 8 to 12 weeks of infection, especially when the infection is asymptomatic. Since MBC populations are maintained for many years, perhaps decades, our findings indicate that MBCs generated by SARS-CoV-2 infection would be available to rapidly generate protective Abs if waning Ab levels were to allow reinfection to occur (31). Notably, three convalescent subjects in our analysis had undetectable RBD-reactive IgG levels but nevertheless had RBD-reactive IgG MBCs. This might reflect MBC production by germinal centers that remained active after recovery from infection (32). The proportion of subjects with MBCs reactive to the HCoVs OC43 and 229E was greater for the convalescent group than for the unexposed group, likely reflecting the increase in levels of S2-reactive MBCs in the convalescent group and cross-reactivity with HCoVs. S2-reactive MBC expansion mediated by SARS-CoV-2 infection could enhance protection against a broad range of coronaviruses (25). The level of N-reactive MBC formation in convalescent subjects was lower than expected given the large number of subjects with high titers of N-reactive IgG, but additional sampling times are required to confirm this observation.

The antigen-specific B cell response to infection and vaccination in humans is characterized by entry into the circulation of recently proliferated class-switched B cells, termed activated B cells (ABCs), which are phenotypically and transcriptionally distinct from ASCs (33). Circulating ABC frequencies peak at 2 to 4 weeks after antigen exposure and have substantially decreased by 3 months. Frequencies of antigen-specific resting MBCs (negative for markers of recent proliferation) increase together with those of ABCs and decrease much more slowly (22, 34). ABCs, like resting MBCs, were activated by the *in vitro* stimulation conditions used in our study to divide and differentiate into ASCs (33). We therefore cannot exclude the possibility that ABC activation contributes, to some degree, to measurement of what we designate MBCs. On the basis of the kinetics of ABC and resting MBC formation and maintenance of immunoglobulin gene clonal lineages in the two populations, Ellebedy et al. (33) suggested that at least a subset of ABCs form resting MBCs. However, the differentiation pathways of ABCs are not well established (34) and the proportion that becomes part of long-maintained MBC populations remains uncertain.

In conclusion, our analysis investigated Ab and MBC immunity to SARS-CoV-2 in unexposed subjects and individuals soon after recovery from SARS-CoV-2 infection. The findings emphasized the novelty of the SARS-CoV-2 S protein RBD in unexposed subjects. However, IgG reactive to the S2 was widespread in unexposed subjects and likely resulted from exposure to HCoVs. Although our approach was unable to directly identify S2-reactive MBCs in the unexposed subjects, we suggest that these cells were present and strongly contributed S2-reactive IgG early in the response to SARS-CoV-2 infection. The IgG response in convalescent SARS-CoV-2 subjects was also strong against the RBD and, less consistently, against the N protein. Importantly, the convalescent SARS-CoV-2 subjects had generated RBD-reactive and S2-reactive IgG MBCs. The RBD-reactive MBCs are likely to provide strong long-term protection if RBD-reactive neutralizing Ab levels wane and reinfection occurs. Additional studies are required to establish the importance of S2-reactive IgG in providing broad anticoronavirus activity and the influence of expanded S2-reactive MBC populations on a *de novo* B cell response to the RBD.

## MATERIALS AND METHODS

### Study participants and clinical samples.

All study participants were recruited at the University of Rochester Medical Center, Rochester, NY, and provided written informed consent prior to inclusion in the studies. The studies were approved by the University of Rochester Human Research Subjects Review Board (protocols 16-0064, 07-0090, and 07-0046) and conducted in accordance with the principles of good clinical practice. A prepandemic cohort of 21 healthy donors (median age, 48 years; interquartile range [IQR], 25 to 70 years) were enrolled from 2011 to 2014 (non-SARS-CoV-2-exposed subjects). A cohort of 20 health care workers (median age, 38 years; IQR, 30 to 52 years) at Strong Memorial Hospital, Rochester, NY, were enrolled in May 2020. The health care workers had not been diagnosed with COVID-19 prior to enrollment. A cohort of 26 nonhospitalized COVID-19 convalescent subjects (9 males and 17 females) (median age, 49 years; IQR, 36 to 63 years) were enrolled in May 2020 and consisted of 22 PCR-confirmed patients and 4 non-PCR-confirmed subjects who were contacts of confirmed cases or displayed COVID-19-like symptoms. The convalescent subjects were sampled 4 to 9 weeks after symptom onset. Symptoms reported (percentages of subjects) were fever (67%), cough (74%), sore throat (48%), stuffy/runny nose (56%), difficulty breathing (52%), fatigue (85%), headache (67%), body aches (67%), nausea/vomiting (19%), and diarrhea/loose stool (41%).

### Recombinant proteins.

RBD and stabilized ectodomain S protein from SARS-CoV-2 (isolate Wuhan-Hu-1) were expressed in-house in HEK293 cells using pCAGGS plasmid constructs kindly provided by Florian Krammer (Icahn School of Medicine at Mount Sinai) (7). Baculovirus-expressed S2 subdomain and HEK293 cell-expressed N protein were obtained from Sino Biological (Chesterbrook, PA) and RayBiotech (Peachtree Corners, GA), respectively. Baculovirus-expressed S proteins from seasonal HCoVs OC43 and 229E were obtained from Sino Biological. In-house HEK293 cell-expressed hemagglutinin from egg-derived H1N1 A/California/7/2009 and TTd (MilliporeSigma, Burlington, MA) were used as noncoronavirus control proteins.

### MBC analysis.

Measurement of levels of antigen-specific MBCs was essentially performed as described previously (22). Briefly, cryopreserved PBMCs were thawed and rested overnight at 37°C in complete medium. Rested PBMCs were stimulated for 6 days at 1 × 10^6^ PBMCs/well in 24-well plates to induce MBC expansion and differentiation into ASCs. The stimulation cocktail consisted of complete medium supplemented with 1 μg/ml R848 (Sigma, St. Louis, MO), 10 ng/ml interleukin-2 (IL-2) (Gibco, Gaithersburg, MD), and 25 ng/ml IL-10 (Stemcell Technologies, Vancouver, Canada). After stimulation, cells were harvested and pelleted by centrifugation. The undiluted supernatant containing Abs secreted by ASCs generated from stimulated MBC precursors (MPAbs) was collected and stored for analysis by ELISA. Supernatants from unstimulated cultures of rested PBMCs were collected to control for Abs produced by preexisting ASCs. Antigen-specific ASCs in the cell pellet (MASCs) were enumerated by ELISpot assay. For each antigen, 300,000 stimulated PBMCs were analyzed by ELISpot assay and the limit of MASC detection was set at 8 spots (MASCs)/10^6^ PBMCs. On the basis of ELISpot assay results, antigen-specific MBCs in peripheral blood were quantified as antigen-specific IgG MASCs as a proportion of stimulated PBMCs. Antigen-specific IgG concentrations in MPAb samples (after subtraction of Ab concentrations in supernatants from the levels seen in unstimulated PBMC control cultures) were also used as a measure of the relative sizes of reactive MBC populations.

### Enzyme-linked immunosorbent assay (ELISA).

Concentrations of Ag-specific serum Abs and MPAbs were measured by ELISA as previously described (22). Briefly, Nunc MaxiSorp 96-well plates (Thermo Fisher, Waltham, MA) were coated overnight with optimized concentrations of antigens. Serially diluted samples were added to blocked plates and incubated for 2 h at room temperature. Alkaline phosphatase-conjugated anti-human IgG (clone MT78; Mabtech, Stockholm, Sweden) and *p*-nitrophenyl phosphate substrate (Thermo Fisher) were subsequently added to detect bound antigen-specific Abs. Absorbance was read at 405 nm after color development. A weight-based concentration method was used to quantify antigen-specific Ab levels in test samples as described previously (22, 35). Sera from healthy donors and convalescent subjects with high titers for test antigens were used to establish human serum standards. The cutoff for assay positivity was set at approximately 2× the mean optical density (OD) value for negative wells.

### Statistical analyses.

The medians (with q1 and q3) were summarized by subject group and compared by the Wilcoxon rank sum test. Spearman correlation analysis was used together with corresponding robust regression models to assess monotonic associations among Ab responses. Multiple-test adjustment was not applied for this explorative study; thus, a *P* value of <0.05 was considered significant for all analyses. Statistical analyses were performed using SAS 9.4 software (SAS Institute Inc, Cary, NC).
